# Perceived Usability of Zoom™ for a Single-Session Telehealth Fall-Risk Screening in Community-Dwelling Older Adults

**DOI:** 10.63144/ijt.2026.6750

**Published:** 2026-06-01

**Authors:** Rolando Lazaro, Amie Marie Jasper, Rania Karim, Carmina (Minnie) Rafael, Eleazar Tayag, Samuel John Montecalvo Uy, Rodiel Kirby Baloy, Arvie Vitente

**Affiliations:** 1 California State University, Sacramento, California, USA; 2 University of St. Augustine for Health Sciences, St. Augustine, Florida, USA; 3 MGH Institute of Health Professions, Boston, Massachusetts, USA; 4 University of St. Augustine for Health Sciences, Miami, Florida, USA; 5 Imperial Home Health, Victorville, California, USA; 6 James A Haley Veterans Hospital, Tampa, Florida, USA; 7 College of St Scholastica, Duluth, Minnesota, USA; 8 Graceland University, Independence, Missouri, USA; 9 Lewis University, Romeoville, Illinois, USA

**Keywords:** Allied health, Occupational therapy, Physical therapy, Screening, System usability, Telerehabilitation

## Abstract

The increased utilization of telehealth services necessitates systematic evaluation of patients’ experiences with technologies, tools, and interventions adapted for the remote delivery of rehabilitation programs. This study examined the usability of Zoom™ as a telehealth platform for delivering a virtual fall-risk screening program in older adults. A convenience sample of community-dwelling older adults participated in a single virtual fall-risk screening session conducted via Zoom. Following the session, participants completed the 9-item System Usability Scale (SUS). Thirty participants (mean age = 71.47 years, *SD* = 5.97) completed the study. The mean SUS score was 76.30 (95% CI [71.47, 82.03]), exceeding the established benchmark of 68 for average usability and approaching the threshold of 80, indicative of excellent usability. Subscale analysis demonstrated that scores for perceived complexity, ease of use, and user confidence exceeded benchmark values, whereas learnability yielded more variable results. Overall, Zoom demonstrated good usability for virtual fall-risk screening in older adults. Participants reported that the platform was easy to use and facilitated confidence, supporting its suitability for remote telerehabilitation screening and intervention delivery.

Usability refers to the extent to which a defined group of users can effectively, efficiently, and satisfactorily engage with an intervention to achieve intended goals (Keenan et al., 2022; Lyon et al., 2021). It encompasses the ease of interaction between the user and the product or system within a specific context or set of conditions (Sandars, 2010). Beyond functionality, usability emphasizes the user experience and the intervention’s contextual adaptability and is a critical determinant of successful implementation (Lyon et al., 2021). Interventions that are easier to use are more likely to be adopted and sustained, resulting in meaningful outcomes. Therefore, assessing usability early and throughout the development and deployment is essential to ensure that an intervention aligns with user needs and performs effectively in real-world settings.

Initially developed by John Brooke to evaluate digital systems, the System Usability Scale (SUS) was designed as a quick and straightforward tool for assessing the perceived usability of a system or product (Brooke, 1996). Research has demonstrated strong correlations between SUS scores and task success in both controlled laboratory and real-world settings (Kortum & Peres, 2014), supporting its broad applicability. The SUS’s widespread use underscores its versatility and reliability in capturing users’ perceptions of system effectiveness, efficiency, and satisfaction, which are core dimensions of usability (Keenan et al., 2022; Lyon et al., 2021). Consequently, the SUS serves as a valuable metric for evaluating user experience across diverse interventions and contexts.

The original version of the SUS consisted of ten items rated on a 5-point Likert scale (from strongly disagree to strongly agree), alternating between positive and negative worded statements. The total score ranges from 0 to 100 and is often interpreted using adjective ratings or letter grades to enhance communication with non-technical audiences. Although the SUS was originally intended to yield a single overall usability score, Lewis and Sauro (2018) expanded its application by developing regression equations from 241 industrial usability studies and surveys, enabling benchmarking of individual items. This approach allows for a more diagnostic understanding of specific attributes. Domain-specific benchmarks, including those developed for mobile health applications, further contextualize SUS results within healthcare (Hyzy et al., 2022). Conventionally, a score of 68 represents average usability, while scores of 80 or higher indicate above average or excellent usability (Bangor et al., 2008).

Over time, researchers have refined the SUS to address concerns related to length, redundancy, and psychometric properties. The SUS has been widely applied to evaluate the usability and accessibility among older adults using mobile health technologies (Khamaj & Ali, 2024; Wang, 2022). A study by De Cola et al. (2020) examined user satisfaction and the effectiveness of teleassistance of physicians, nurses, and social healthcare services for older adults with frailty. Similarly, Lin et al. (2009) used the SUS to assess older adults’ ability to interact with a touchscreen-enabled Personal Education Program (PEP) developed with advanced practice registered nurses. More recent studies have applied the SUS to evaluate an e-learning platform supporting electronic personal health record (ePHR) use (Perotti et al., 2025) and a tablet-based e-coach promoting nutrition and physical activity in older adults (Happe, 2023).

The increased utilization of telehealth is evident across a wide range of healthcare practice settings, spanning multiple disciplines including rehabilitation, nursing, occupational therapy and other allied health professions. Growing evidence demonstrates high patient satisfaction with telehealth across these fields, including physical therapy (Tenforde et al., 2020). Research further suggests that telehealth-delivered interventions can be as effective, or even superior to, an in-person therapy session for a variety of conditions, including osteoarthritis, low-back pain, hip and knee replacements, multiple sclerosis, and in cardiac and pulmonary rehabilitation (Seron, 2021).

Additionally, studies across rehabilitation and geriatric care indicate that certain balance and gait outcome measures, such as Timed-Up and Go (TUG), and all the components of the Stopping Elderly Accidents, Deaths & Injuries (STEADI) Fall Risk Assessment can be reliably administered via telehealth for older adults (Jasper et al., 2023, 2025; Karim et al., 2023). Successful remote assessment, however, depends on the participants having appropriate electronic devices, reliable internet connectivity, and sufficient technological proficiency with camera setup (Jasper et al., 2023, 2025; Karim et al., 2023). As telehealth continues to expand, evaluating patients’ experiences with remote technologies and interventions is critical for optimizing the delivery and outcomes of rehabilitation programs.

The use of telehealth across healthcare disciplines represents a significant advancement in healthcare delivery (Wosik et al., 2020). These technologies not only improve access to care but also enhance patient engagement and outcomes. Assessing user perceptions of virtually delivered services is essential for optimizing virtual care models and ensuring their effectiveness in supporting health and safety among older adults. This study aims to evaluate the perceived usability of Zoom^TM^ as a telehealth platform for delivering a virtual fall screening program among community-dwelling older adults.

## Methods

As part of a study assessing the concurrent validity and reliability of the CDC’s STEADI delivered both in-person and via remote supervision for community-dwelling older adults, participants completed the SUS. Conducted from August to December 2023, the study recruited a convenience sample of older adults and was approved by the Institutional Review Board at the University of St. Augustine for Health Sciences (IRB Number UR813380). Written informed consent was obtained for all participants.

Participant eligibility was determined via an online screening form created in Microsoft Forms. Inclusion criteria were: age 65 years or older; ability to follow instructions in English; perform sit to stand with or without upper extremity support; walk 20 feet unassisted by another individual with or without assistive device; stand for two minutes or longer without dizziness; score of six or greater on the Short Portable Mental Status questionnaire; access to high-speed internet (4G, 5G, internet cable) and videoconferencing-enabled device (laptop, tablet, or cellphone that has video and audio); ability to operate or have assistance operating a laptop, tablet or cellular phone, and the telehealth platform; and full weight-bearing capability on both lower extremities. Exclusion criteria included unstable medical conditions (heart, lungs, or metabolic disorders), a history of falls of syncopal origin, low vision or complete loss of vision, or complete loss of hearing.

Once eligibility was confirmed by the principal investigator, a testing session was scheduled with the participant and Raters 1 and 2. Prior to the sessions, Rater 1 emailed the participant the Zoom link, instructions for use, session preparation details, informed consent, and a link to the fall risk factors checklist containing three key clinical screening questions. Participants then completed a single-session test of the TUG, 30s-CST, and 4-stage balance test in their own environment, using their own videoconferencing device and internet. The participant was responsible for connecting to the established Zoom link and repositioning the camera as requested by Rater 2, who provided all assessment instructions. Rater 1 observed but only intervened for immediate safety concerns and did not assist with technology or setup. Caregivers, if present, were allowed to provide support. Immediately after the session, participants received a digital copy of the 9-item SUS via email with instructions to complete and return it to the principal investigator.

### Instrument

This study used a 9-item version of the SUS to reduce survey burden and cognitive load, which may improve completion rates and reduce measurement error from fatigue. Previous research has shown that omitting a single item has minimal impact on the total score (Lewis & Sauro, 2017). Intercorrelations between the 9-item and the full SUS are extremely high, indicating that the shortened version captures the same construct as the full instrument (Lewis & Sauro, 2017). In this study, Item 6 of the original SUS survey, “I thought there was too much inconsistency in this system,” was removed as it was not applicable to a single-session Zoom interaction. Procedures for scoring the 9-item SUS are described in the Data Analysis section. Both the total SUS score and the domain-specific attributes of the 9-item scale were computed as follows:

*Perceived Complexity* was primarily captured through Item 2: *“I found the system unnecessarily complex,”* and Item 7: *“I found the system very cumbersome to use.”* High scores on these items indicate that users perceive the system as simple and not overly complicated. Complexity contributes to the overall usability construct by highlighting potential barriers to efficiency and user satisfaction.

*Perceived Learnability* is a distinct but closely related component of usability. Within the 9-item SUS, Items 4 *(“I think that I would need the support of a technical person to be able to use this system”*) and 6 *(“I would imagine that most people would learn to use this system very quickly”*) were used.

*Ease of use* was reflected in Item 3: *“I thought the system was easy to use.”* In healthcare applications where efficiency and user-friendliness are crucial for adoption, the ease-of-use dimension has proven especially valuable for comparing electronic health records, telehealth tools, and mobile health applications (Hyzy et al., 2022).

*Confidence* in using a system was captured by Item 8 (“*I felt very confident using the system”*). Confidence serves as an indicator of user self-efficacy and perceived competence with the system, linking usability with psychological readiness for adoption (Lewis, 2018). In healthcare, where clinicians’ confidence in interacting with digital systems influences workflow and patient safety, this component is particularly relevant (Cahill et al., 2025; Mol et al., 2020).

Evidence across multiple domains consistently demonstrates high internal consistency of the SUS, with Cronbach’s alpha values typically near .90, supporting its utility as a reliable global measure (Lewis, 2018; Sauro, 2009). Studies conducted in healthcare environments, such as evaluations of clinician-facing digital interventions (e.g., internet-based cognitive behavioral therapy platforms), have reported similarly strong reliability indices, reinforcing the stability of the SUS score among professional users (Mol et al., 2020).

Construct validity of the SUS has been supported by correlations with other measures of usability and satisfaction, as well as with global ratings of user experience (Lewis, 2018; Peres, 2013). Within medical device studies, the SUS has shown moderate convergence with device-specific scales, suggesting that it captures core elements of usability (Ghorayeb et al., 2023). In terms of criterion validity, the SUS has demonstrated sensitivity to differences in usability across systems and products, including healthcare applications such as clinical decision support tools, telehealth platforms, and electronic health record modules (Cook & Beckman, 2006).

### Data Analysis

Demographic and participant characteristics were analyzed using descriptive statistics to summarize the sample composition. Frequencies and percentages were calculated for categorical variables such as sex, platform type (smartphone, tablet, laptop), living arrangement, caregiver assistance, and use of assistive devices. Measures of central tendency and dispersion, including means, standard deviations, and ranges, were computed for continuous variables such as age, cognitive status (via Short Portable Mental Status Questionnaire scores), and balance confidence (via Activities-specific Balance Confidence [ABC] scores). These descriptive analyses provided a profile of the study sample and contextualized usability outcomes relative to participant characteristics. All statistical analyses were performed using IBM SPSS Statistics (version 30)

The usability of Zoom for the telehealth assessment was evaluated using a 9-item SUS. SUS scores are computed using a 5-point Likert scale, where 1 represents strongly disagree, and 5 represents strongly agree. In our 9-item SUS, Items 1, 3, 5, 6, and 8 were the positively worded statements, while Items 2, 4, 7, and 9 were negatively worded. For the positively worded statements, 1 point was subtracted for the user’s Likert ratings, while in the negatively worded statements, the user’s Likert ratings were subtracted from 5. The sum of the adjusted item scores was divided by 36, then multiplied by 100 to obtain the final score, which reflects a 0-100 scale.

We selected four attributes (*Perceived Complexity, Perceived Learnability, Ease in Use, and Confidence in Use*) that were most applicable among the five-item level benchmarks described by Lewis (2018) and Lewis and Sauro (2018). We did not select the attribute related to *Consistency in use* because this corresponded to the original Item 6 (“I thought there was inconsistency in the system”), which we did not include in our 9-Item SUS. Mean scores were obtained for each attribute. We likewise used the study by Lewis and Sauro (2018) to compare the scores obtained to the benchmarks reported in that study. Internal consistency reliability of the 9-item SUS used in this study was assessed using the Cronbach’s alpha coefficient. Cronbach’s alpha evaluates the degree of interrelatedness among items within a scale, serving as a measure of the instrument’s internal consistency. Values above 0.70 are generally considered acceptable, while those exceeding 0.80 indicate good to excellent reliability for psychological and usability measures. We conducted reliability testing on the full scale, the positively worded questions (Items 1,3,5,6 and 8), and the negatively worded items (Items 2,4,7 and 9)

## Results

### Participant Characteristics

Out of 43 participants, 42 met the inclusion criteria. A convenience sample of 30 participants with a mean age of 71.47 years (*SD* = 5.97 years, range = 65 – 88 years) provided consent to participate and complete the tests. Table 1 summarizes the demographic information of the participants. There were twenty females (66.67%) and ten males (33.33%). Twenty (66.67%) participants used their smartphones for videoconferencing, four (13.33%) used tablets while six (20%) used laptops. Regarding cognition, 29 participants (96.7%) had normal cognitive functioning while one participant (3.3%) had mild cognitive impairment based on the Short Portable Mental Status questionnaire scores. The mean Activities-specific Balance Confidence scale (ABC) score was 88.51 (range 27 – 100) with 24 (80%) participants considered to have a high level of physical functioning, while 4 (13.3%) and 2 (6.7%) were noted to have moderate and low levels of functioning respectively. Six participants (20%) received caregiver assistance for telehealth setup. All participants lived in urban areas. (Table 1)

### System Usability Scale Questionnaire

The full scale of SUS shows strong internal consistency, with a Cronbach’s alpha of 0.76. The subscales for the positively worded questions only and the negatively worded questions only had Cronbach’s alpha of 0.82 and 0.80, respectively. The average SUS total score of 76.30 (*SD* =15.35) 95% CI [70.57, 82.03] exceeded the benchmark of SUS 68 for an average user experience. Regarding components, the virtual fall screening program exceeded the benchmark of SUS=68 (average experience) for perceived complexity (Items 2 and 7) while perceived ease of use (Item 3) and confidence in use (Item 8) exceeded the benchmark of SUS 80 (above average experience). Perceived learnability had mixed results, with item 6 meeting the SUS 68 benchmark while Item 4 did not. Table 2 summarizes the results of the total and component scores compared to the benchmarks.

In terms of individual items, four items on the scale exceeded the average user experience (SUS=80), while three exceeded the average user experience (SUS=68). Items 4 and 9 did not exceed the SUS benchmark. (Table 3)

## Discussion

The 9-item System Usability Scale (SUS) served as an effective, psychometrically sound instrument for quantifying the usability of the Zoom platform in telehealth-based fall-risk assessments among community-dwelling older adults. The mean SUS score of 76.30 (*SD* = 15.35) exceeded the established benchmark of 68, indicating a “good” level of usability (Bangor et al., 2008; Brooke, 1996). This indicates that participants were generally able to navigate the platform effectively to complete the required virtual assessment tasks. Further analysis of SUS into the component domains of complexity, learnability, ease of use, and confidence enhanced the precision of data interpretation and enabled identification of specific usability strengths and limitations within the telehealth context. This multidimensional analytic approach aligns with established recommendations in usability research that advocate for the examination of data to inform targeted system refinement and implementation planning (Kortum & Peres, 2014; Lewis & Sauro, 2018).

Our study found that the mean total usability score, perceived complexity, perceived ease of use, learnability, and confidence either met or exceeded their respective benchmarks. This study highlights that with appropriate guidance and caregiver support, older adults can successfully navigate virtual platforms like Zoom for health assessments. Clinicians who screen for risk for falls should consider telehealth a viable option for delivering virtual fall-risk screenings and interventions, acknowledging that while there may be an initial learning curve, participants generally adapt and gain confidence with continued use.

The study’s use of a 9-item adaptation of the SUS reflects an intentional methodological modification aimed at mitigating cognitive and response burden in a cohort of older adults. Prior validation studies have demonstrated that omitting a single item does not significantly affect internal consistency or construct validity, with correlations exceeding 0.95 relative to the original 10-item scale (Bangor et al., 2009; Lewis & Sauro, 2017). In this study, internal reliability indices were found to be robust and consistent with reliability coefficients reported in related literature on SUS healthcare technology applications.

The obtained SUS scores align closely with those reported in healthcare usability studies. Digital systems designed for clinician use, such as electronic health records (EHRs), computerized decision support tools, and telemonitoring dashboards, commonly yield lower SUS values (55–68), reflecting higher interactional complexity and integration demands (Finstad, 2006; Peres et al., 2013). Conversely, patient-facing telehealth and mobile health (mHealth) applications typically yield SUS scores of 70–80, indicating generally favorable usability, particularly when interactional tasks are narrowly defined and appropriately supported (De Cola et al., 2020; Happe et al., 2023; Perotti et al., 2025). The present mean score of 76.3 thus positions the evaluated platform in the upper quartile of the usability continuum for digital health systems, corroborating previous findings that older adults can effectively use telehealth tools when adequate support and intuitive interface design are provided (Lin et al., 2009; Wang et al., 2022).

Analysis of the SUS attributes yielded further insights. Participants reported particularly high scores in perceived ease of use and confidence, suggesting that once connected, they were able to operate Zoom with minimal difficulty and high self-efficacy. The complexity scores were significantly lower than the other attributes of the SUS. This could be due to the increased cognitive and sensorimotor load on the participant, coupled with the environmental and contextual setup requirements. Within this dynamic, patients may be required to perform challenging tasks as part of the actual plan of care, while at the same time being mindful that the movements and responses are adequately focused and visually framed to allow the remote clinician an adequate view to make appropriate decisions. This scenario is particularly applicable across rehabilitation and other movement-based healthcare disciplines, such as occupational therapy and physical therapy, where constant assessments of changes in dynamic movements, and therefore camera views, are critical, as opposed to other remote healthcare interactions where an adequate static view of the face of the clinician and patient is sufficient for an effective interaction. These findings echo prior reports that environmental and contextual barriers, rather than intrinsic system usability, often constitute the most salient limitations in telehealth deployment for older populations (Tenforde et al., 2017). However, usability may be influenced by participant familiarity with technology. Individuals who are more technologically experienced may find platforms like Zoom easier to navigate, which should be considered when interpreting the findings.

The pattern of results underscores that system-level usability is satisfactory, and that targeted environmental and instructional interventions (e.g., setup checklists, caregiver facilitation, or use of device mounts) may further optimize user experience and data fidelity. This also highlights the importance of sufficient environmental setup to ensure that results are valid, reliable, and meaningful.

### Limitations

Although Zoom demonstrated good overall usability for telehealth fall-risk screening in older adults, several limitations should be considered when interpreting the results of this study. First, the virtual fall screening, using Zoom as the telehealth platform, was conducted as a single session. The effect of repeated sessions is unknown, especially in domains such as perceived complexity and learnability. Also, the convenience sampling method could be a source of participation bias. Future studies can include a larger sample size stratified based on prior telehealth knowledge and experience. Some participants may have been relatively “tech-savvy,” which could have influenced usability scores. Future studies should stratify participants based on prior technology experience. We also had a lack of representation of participants from rural areas, which is a subpopulation that can benefit from improved access to services such as a fall risk assessment. Lastly, since there are two groups of users in this telehealth virtual fall-risk screening session (namely the participants and the physical therapists), it would be worthwhile to explore the perceived usability from the perspective of the physical therapists or other individuals conducting the screening. Future research can utilize a mixed-method design to identify opportunities for improving user experience from the perspectives of the stakeholders involved in the activity.

## Conclusion

Zoom is a viable platform for telehealth delivery across multiple rehabilitation disciplines. Our findings affirm that the 9-item System Usability Scale (SUS) provided a reliable, comprehensive measure of user interaction quality within the telehealth environment, effectively capturing both overall satisfaction and domain-specific aspects of system performance. The acceptable total score, coupled with strong component results, underscores the suitability of the Zoom platform for remote clinical applications among older adults. While usability is high, onboarding and training may improve learnability among less experienced users of digital technology.

## Figures and Tables

**Table 1:** Participant Characteristics (N = 30)

Characteristics	Value	Frequency	Percentage (%)
Age	65–74 years	23	76.7
	75–84 years	5	16.7
	85 years and older	2	6.7
Sex	Female	20	66.7
	Male	10	33.3
Platform	Smartphone	20	66.7
	Tablet	4	13.3
	Laptop	6	20.0
Short Portable Mental Status Score	Normal Cognitive Functioning	29	96.7
	Mild Cognitive Impairment	1	3.3
Activities-Specific Balance Confidence Scale	High level of physical functioning (above 80)	24	80.0
	Moderate level of physical functioning (51–79)	4	13.3
	Low level of physical functioning (below 50)	2	6.7
Lives alone	Yes	24	80.0
	No	6	20.0
Caregiver assistance for telehealth setup	Yes	6	20.0
	No	24	80.0
Assistive device use	Yes	6	20.0
	No	24	80.0

**Table 2:** Total Scores, Component Scores and Benchmarks

	Score (*SD*)	Corresponding items	Benchmark	Benchmark descriptor of user experience	Benchmark achieved?
Total score	76.30 (15.35)	Items 1–9	68	Average	Yes
Perceived Complexity	2.03 (1.50); 1.90 (1.30)	Items 2 and 7	68 (less than or equal to 2.44/2.25)	Average	Yes
Perceived Learnability	4.17 (0.87); 4.17 (0.88)	Items 4 and 6 68 (greater than or equal to 4.45)	Average	No	
Ease in Use	4.37 (0.81)	Item 3	80 SUS 80 (greater than or equal to 4.34)	Above average	Yes
Confidence in Use	4.47 (0.78)	Item 8	80 SUS 80 (greater than or equal to 4.45)	Above average	Yes

*Note.* SD, standard deviation

**Table 3:** Individual Items of the 9-item SUS (N = 30)

Question	Mean (*SD*)	Item Benchmark
Q1. I think I would like to use this system frequently	4.03 (1.07)	SUS = 80 (greater than or equal to 3.80)
Q2. I found the system unnecessarily complex	2.03 (1.50)	SUS = 68 (less than or equal to 2.44)
Q3. I thought the system was easy to use	4.37 (0.81)	SUS = 80 (greater than or equal to 4.24)
Q4. I think I would need the support of a technical person to be able to use the system.	2.5 (1.55)	SUS = 68 (less than or equal to 1.85)
Q5. I found the various functions in this system were well integrated.	4.27 (1.05)	SUS = 80 (greater than or equal to 3.96)
Q6. I would imagine that most people would learn to use this system very quickly.	4.17 (0.88)	SUS = 68 (greater than or equal to 3.71)
Q7. I found the system very cumbersome to use.	1.90 (1.30)	SUS = 68 (less than or equal to 2.25)
Q8. I feel confident using the system.	4.47 (0.78)	SUS = 80 (greater than or equal to 4.25)
Q9. I needed to learn a lot of things before I could get going with this system.	2.40 (1.25)	SUS = 68 (less than or equal to 2.09)

*Note.* SD, standard deviation; Q, question; SUS, system usability scale
